# Recent Progress in Plasmonic based Electrochemiluminescence Biosensors: A Review

**DOI:** 10.3390/bios13020200

**Published:** 2023-01-29

**Authors:** Cheng Ma, Zhichen Zhang, Tingting Tan, Jun-Jie Zhu

**Affiliations:** 1 School of Chemistry and Chemical Engineering, Yangzhou University, Yangzhou 225002, China; 2 State Key Laboratory of Analytical Chemistry for Life Science, School of Chemistry and Chemical Engineering, Nanjing University, Nanjing 210023, China

**Keywords:** plasmonic, electrochemiluminescence, biosensors, microscopy, nanomaterials

## Abstract

Electrochemiluminescence (ECL) analysis has become a powerful tool in recent biomarker detection and clinic diagnosis due to its high sensitivity and broad linear range. To improve the analytical performance of ECL biosensors, various advanced nanomaterials have been introduced to regulate the ECL signal such as graphene, gold nanomaterials, and quantum dots. Among these nanomaterials, some plasmonic nanostructures play important roles in the fabrication of ECL biosensors. The plasmon effect for the ECL signal includes ECL quenching by resonant energy transfer, ECL enhancement by surface plasmon resonance enhancement, and a change in the polarized angle of ECL emission. The influence can be regulated by the distance between ECL emitters and plasmonic materials, and the characteristics of polarization angle-dependent surface plasmon coupling. This paper outlines the recent advances of plasmonic based ECL biosensors involving various plasmonic materials including noble metals and semiconductor nanomaterials. The detection targets in these biosensors range from small molecules, proteins, nucleic acids, and cells thanks to the plasmonic effect. In addition to ECL biosensors, ECL microscopy analysis with plasmonic materials is also highlighted because of the enhanced ECL image quality by the plasmonic effect. Finally, the future opportunities and challenges are discussed if more plasmonic effects are introduced into the ECL realm.

## 1. Introduction

**Figure 1:**
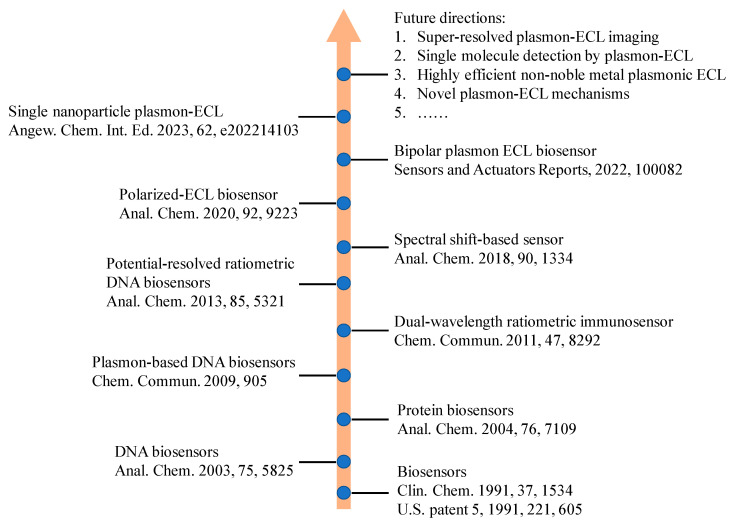
A roadmap for the progress of ECL biosensors and its future directions.

As a member of the luminescence family, electrochemiluminescence (ECL) is a special form of chemiluminescence induced by redox reactions among electrogenerated high-energy radicals [1]. Because ECL does not depend on external light excitation, the adverse influence of autophotoluminescence and light scattering in analytical measurements can be avoided [2,3,4]. Compared with chemiluminescence techniques, ECL possess better spatial and temporal controllability for emission regions and time [5,6,7,8,9,10,11]. Therefore, more and more ECL biosensors have been developed due to its low light background, high sensitivity, and broad dynamic range [12,13,14,15]. The key point in the design of an ECL sensor is to optimize the performance of the luminophore or electrode substrate for the sake of enhanced ECL sensitivity [2,3,4,16,17,18,19,20]. Recently, various signal amplification methods have been proposed to improve the analytical properties of ECL sensors [4,12,13,16,21,22]. These approaches included using enzyme-mediated amplification, DNA-mediated amplification, new electrochemical catalytical substrate, and nanomaterial-assisted amplification [23,24]. Enzyme-mediated amplification used various enzymes to catalyze biochemical reactions and amplify the targets. Its merits include the flexible choice from abundant enzymes, high signal enhancement efficiency, and high selectivity. However, the vulnerable natural activity of enzymes can be more easily destroyed by harsh environments. DNA-mediated amplification is a kind of enzyme-free amplification technique by using DNA as the tool to improve the ECL signal. Thus, DNA-mediated amplification has better stability in a long term. However, most DNA amplification strategies rely on the hybridization of double stranded DNA, which is affected by the environment temperature. Electrochemical catalytical substrates can promote the electron transfer and surface area of the electrode, thereby enhancing ECL signals in some situations. Nanomaterial-assisted amplification can use nanomaterials as carriers of ECL emitters. Numerous ECL luminophores can be incorporated to carrier frameworks to provide much stronger ECL intensity for ultrasensitive biosensors. However, the uneven and heterogeneous nanocarriers may cause ECL signal deviation. In addition, luminophore leak and poor conductivity of the nanocarrier are problems to be solved. So far, many ECL-based biosensors with high sensitivity and selectivity have been fabricated to detect important analysts in the fields of environmental science, clinical diagnostics, food and water testing, and biowarfare agent detection [10]. In addition, ECL microscopy has become increasingly popular in analytical science due to its spatial and temporal resolution that has facilitated single-cell and single-particle analysis [25,26]. To briefly show the development history of ECL biosensors, we illustrated a roadmap for the progress of ECL biosensors and its future directions (Figure 1).

Surface plasmon resonance (SPR) occurs at the interface between a metal phase and a dielectric material [27,28]. After being excited by external optical radiation or in the presence of strong static electric and magnetic fields, a surface plasmon of the electrons at the surface of materials showed a collective oscillation wave, which propagated along the boundary between the dielectric and the metal. In addition, the SPR phenomenon was observed in not only noble metals but also in semiconductors such as MoS_2_ [29,30]. The plasmonic effect has been widely used in many important fields such as photocatalysis, energy storage, and biosensors [31,32,33]. Localized surface plasmon resonance (LSPR) is a plasmon that oscillates locally around the nanoparticle. Therefore, LSPR is sensitive to changes in local dielectric environment. The effect of localized surface plasmon resonance (LSPR) has been well used in the detection of several biomolecules, including creatinine, cardiac troponin I, alanine aminotransferase, acetylcholine, cholesterol, uric acid, and p-cresol [34,35,36,37,38,39,40]. Optical emissions can be enhanced in the presence of the SPR effect, such as surface enhanced Raman scattering spectroscopy, total inner reflection, fluorescence, and chemiluminescence. Particularly, the molecular Raman scattering cross-section has an improved order-of-magnitude due to the excitation of collective electron oscillation of metal and semiconductor nanostructures [41,42,43,44]. Although surface enhanced Raman spectroscopy used noble metal nanostructures to enhance the Raman scattering signal from nearby molecules, an extrinsic probe is required for surface enhanced Raman spectroscopy. In fluorescent analytical methods, the distance between luminophores and metal nanostructures can cause the switch from fluorescence quenching to fluorescence enhancing [45,46]. The basic mechanism is the competition between the non-radiative transition in Forster resonance energy transfer and electromagnetic field enhancement. When a chromophore is in the evanescent field near metal surface, the large enhancement of fluorescence can occur where plasmon resonance reaches its maximum. However, photobleaching and photodamage are the roadblocks for plasmon-enhanced fluorescence methods. Recently, the plasmonic coupling and electromagnetic interaction exhibited tunable optical properties for ECL through regulating the distance between ECL emitters and plasmonic nanostructures. Due to the absence of an external light source, the ECL reactions at different plasmonic interfaces show different electrochemical rates and enhancement factors without the issue of photobleaching. Some studies involving ECL microscopy provided visualized evidence to characterize the plasmonic enhancement sites at the sub-particle level [47,48,49]. The surface plasmon also caused ECL polarization due to electron oscillation in the noble metals [50]. As a result, the ECL analytical performance can be improved if the polarized ECL signal can be used as the detection signal.

**Figure 2:**
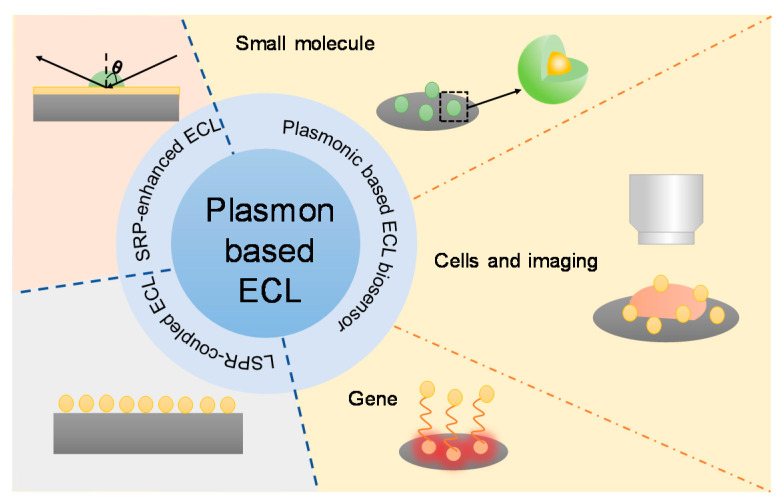
A schematic diagram illustrating the content of this review.

Therefore, this review overviews recent ECL biosensors as well as microscopy to determine whether performance is improved by plasmonic effects. The content of this review is briefly shown in Figure 2. The first part discussed the fundamentals of the plasmon coupled ECL phenomenon, emphasizing the mechanisms of plasmonic ECL. The coupling of ECL and plasmon can cause ECL enhancement, quenching, or a change in the polarization angle. The second part introduced the recent advanced biosensors with the aids of plasmonic materials. The detection targets included small molecules, genes, proteins, cells as well as other important biomarkers. In addition to conventional ECL intensity measurements, ECL microscopy has also been used to acquire spatial and temporal resolution in plasmon-based ECL events. We also recommend readers interested in plasmonic ECL to refer to previous excellent reviews that emphasized gold nanoparticles or resonance energy transfer phenomena [21,22,51].

## 2. Fundamentals of Plasmon-Coupled ECL

**Figure 3:**
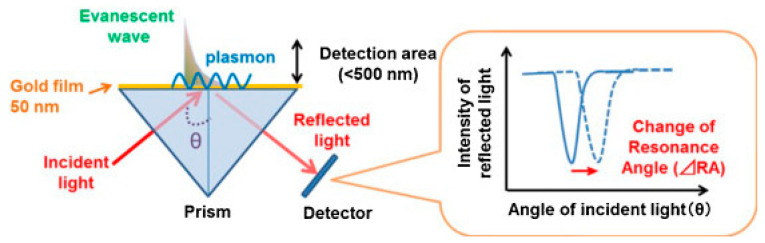
Schematic description of SPR sensor. Reprinted with permission from *Sensors*, Copyright 2014, MDPI [54].

Plasmon is the collective oscillation of conduction electrons in metals or semiconductors. Specifically, many noble metals and semiconductors have surface plasmon resonance properties. When ECL emission sites are very close to surface plasmons, the surface plasmons strongly interact with light. As a result, the ECL emission can be quenched or enhanced by the plasmonic effect, depending on the distance between the ECL emitter and plasmonic materials, as well as other factors. In addition, the polarization angle of ECL emission can be changed by the plasmonic effect. So far, the ECL emission can be regulated by two plasmon resonance effects including surface plasmon resonance (SPR) and localized surface plasmon resonance (LSPR). In terms of SPR, the most typical and widely used setup is the Kretschmann configuration, which uses thin gold film coated glass as the substrate. When the optimal beam is irradiated, metal substrates show strong surface plasmonic polarization (Figure 3). The resonance angle changed with the refractive index near gold film, which can be influenced by biomolecule recognition on gold film. Compared with SPR, LSPR is generated on metal nanoparticles under light irradiation, which caused collective electron charge oscillations in metallic nanoparticles. In addition, the plasmonic effect is highly localized at the nanoparticles and decays quickly away from the nanoparticle. Therefore, many ECL biosensors used the distance-dependent LSPR effect to probe structure changes of DNA or a sandwiched immunoassay. For the plasmon coupled ECL system, SPR-based properties are highly dependent on the nanostructures of plasmonic materials. Metallic nanostructure arrays and porous metal membranes were wildly used as the source of plasmon because of their superior plasmonic properties and controllability via strategies of assembly and morphological regulation. The metallic nanostructures used in plasmon coupled ECL can improve ECL performance through the improvement of electrical conductivity, electro-catalytic activity, electromagnetic field enhancement, and so on. Xia et al. investigated the gold-coated polydimethylsiloxane chip by a stamping and spraying process [52]. The plasmonic gold microwells significantly enhanced ECL intensity for the analysis of intracellular glucose. Elena et al. has reported that ECL emission was related to the effective surface area of the nanoporous gold electrode [53]. Therefore, the nanoporous gold electrode possessed brighter ECL emission compared with the flat gold electrode. All in all, plasmonic materials with stable plasmonic performance, high specific surface area, multiple active sites, and good electrochemical properties played a key role in plasmon coupled ECL.

### 2.1. SPR-Coupled ECL

To provide a deep understanding of the interaction between SPR and ECL, Dinel et al. reported the first combination of SPR and ECL techniques to investigate the interfacial adsorption and energy transfer processes between ECL and the plasmonic substrate [55]. In this work, the ECL evolution on a classic Au chip for SPR measurements was employed under the irradiation of external LED light for the excitation of SPR. Tween 80 was used as a protective layer on the gold film. The real-time monitoring capabilities enabled studies on the impact of plasmon resonance on the ECL process of Ru(bpy)_3_^2+^. Various control experiments demonstrated that SPR and ECL can be detected simultaneously without interference. ECL intensity was quenched in the presence of illumination of the SPR chip because the activated plasmon reduced the probability of radiative relaxation of the excited luminophore. This phenomenon indicated that ECL emission occurred very close to the electrode surface after being modified by Tween 80. The combination of SPR and ECL provided fundamental insights into the mechanism of interfacial processes involving electrochemical reactions and electrode passivation.

**Figure 4:**
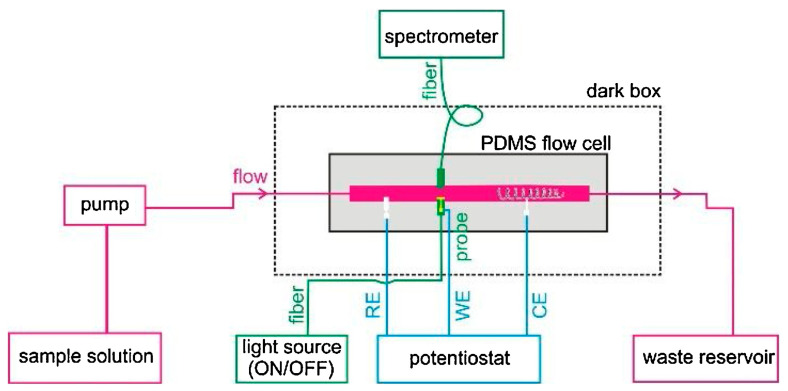
Assembly schematic diagram of the flow injection fiber optic ESPR device. Reprinted with permission from *Sensors and Actuators B: Chemical*, Copyright 2020, Elsevier [56].

The requirement of sophisticated apparatus and complex optics of the conventional Kretschmann prism-based SPR setup inhibits applications in sensing and online monitoring. To provide an easier configuration, Yu et al. designed a flow injection ESPR device (Figure 4) through a new electrochemical SPR conductive fiber where a gold nanohole array film was integrated [56]. This configuration enabled direct collection of the plasmonic response in transmission mode. Considering the portability and low cost of the fiber-based probe, the co-reactant-based ECL system, including Ru(bpy)_3_^2+^ and TPrA, was tested to provide interfacial SPR information and mechanistic insights into the ECL process. The SPR signal directly revealed the oxidation reaction of TPrA in the vicinity of the electrode surface due to the change in the local refractive index.

### 2.2. LSPR-coupled ECL

**Figure 5:**
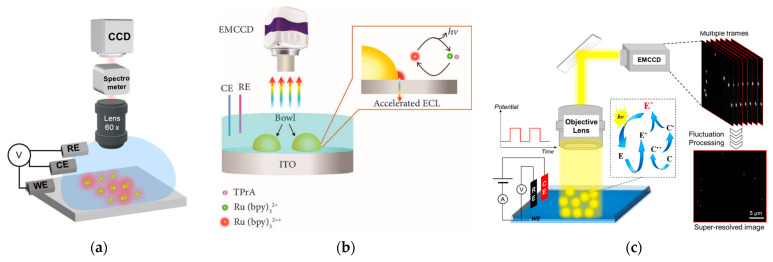
(**a**) Schematic illustration of ECL microscopy for imaging an Au NP array with a 5 μm interval. Reprinted with permission from *Angewandte Chemie*, Copyright 2022, Wiley-VCH [59]. (**b**) Schematic representation of the electrochemiluminescence imaging. The luminophore, Ru(bpy)_3_^2+^, and co-reactant, TPrA, are oxidized at the heterogeneous interface between the microbowls and the ITO supporting electrode with the aid of an enhanced electric field, generating the excited state Ru(bpy)_3_^2+*^. The accelerated ECL emission is produced during the relaxation of Ru(bpy)_3_^2+*^ back to the ground state. Reprinted with permission from *RESEARCH*, Copyright 2021, AAAS [47]. (**c**) Schematic illustration of the ECLM system for single-particle imaging and basic principle of SRRF analysis of multiple images. Reprinted with permission from *Journal of the American Chemical Society*, Copyright 2021, American Chemical Society [48].

In addition to SPR generated on two-dimension gold film, LSPR generated on zero-dimension or one-dimension gold nanomaterials was more commonly used to regulate ECL emission. The enhanced ECL efficiency by LSPR was determined in the presence of citrate-coated gold nanoparticle aqueous solutions [57]. The classic ECL reactions between Ru(bpy)_3_^2+^ and oxalate were tested in an aqueous solution containing 5 nm gold nanoparticles. The SPR band of gold nanoparticles solution showed a red-shift in the presence of Ru(bpy)_3_^2+^, which indicated the nanoparticle aggregation and promoted the SPR-ECL coupling effect with 620 nm ECL emission of Ru(bpy)_3_^2+^. The integrated ECL emission was increased by approximately 3-fold compared with its value in the absence of gold nanoparticles. Although gold nanomaterials are known to be able to improve the electrochemical rate, the direct observation of ECL enhancement at single gold nanomaterials was not realized yet. Therefore, Pan et al. discovered that ECL intensity was enhanced with the increase in the size of gold nanoparticles with ECL microscopy [58]. Thanks to the spatial and temporal resolution of ECL microscopy, the local ECL signal from different gold nanoparticles with time elapsing were recorded in a high throughput manner. Two kinds of gold nanoparticles, namely, electrodeposited and pre-synthesized gold nanoparticles, both showed ECL enhancement, but electrodeposited gold nanoparticles showed a narrow ECL intensity distribution. In addition, ECL microscopy was used to study the plasmon-enhanced ECL at the level of a single nanoparticle based on the ordered array of Au NPs (Figure 5a) [59]. Thanks to the establishment of the precise location and a high throughput platform, LSPR led to the increased ECL by large Au NPs (>80 nm) in the Ru(bpy)_3_^2+^/TPrA system, which was related with the Au NP configuration and sizes. The coupled Au NPs demonstrated higher ECL strength compared with the uncoupled ones, which was consistent with numerical simulations. This plasmon-enhanced ECL imaging strategy can be used in single-particle electrocatalysis in the future.

Spectral overlap between nanoparticle scattering and ECL emission was considered as a prerequisite to generate surface plasmon coupling ECL. Although many reports based on ensemble spectroscopy demonstrated that plasmonic nanostructures can act as signal boosting antennas to amplify ECL, single-nanoparticle heterogeneities and the structure–function relationship remain uncovered. To provide insight into the spectral overlap at the single-particle level, Heiderscheit et al. investigated the influence of nano-emitter spectral overlap under the microscopic view [60]. It was found that those nanoparticles with a larger spectral overlap between scattering and ECL showed stronger ECL enhancement. The measurement at the single particle level excluded the influence from adjacent particles and the average effect. The ECL intensity can be increased up to 10-fold at the maximal spectral overlap using either gold nanoparticles or gold nanotriangles. In addition to spectral overlap, Cui et al. found that an enhanced electric field also boosted the ECL signal. They fabricated a heterogeneous interface by loading bowl-like gold particles on the indium tin oxide (ITO) substrate electrode (Figure 5b) [47]. The heterointerface between the gold bowl and ITO produced an enhanced electric field, which caused a bright ECL ring around the gold bowl. COMSOL simulations showed similar results with the captured ECL patterns from EMCCD. This work suggested that the heterogeneous distribution of the electric field effect could serve as a new mechanism to accelerate the electrocatalytic reaction rate as well as the relevant ECL intensity in biosensors.

Although isolated noble metal particles have been often used to test the surface plasmonic ECL effect, periodic particle arrays with surface lattice resonances can also regulate the luminescence properties in many cases. Heiderscheit et al. further demonstrated that the hexagonally packed arrays of gold nanodisks enhanced the ECL of Ru(bpy)_3_^2+^ and TPrA [61]. Both theoretical and experimental results confirmed that the nanodisk spacing and measurement geometry affected the ECL enhancement factors. After measuring the ECL intensity and spectrum under all lattice spacings, a 24-fold ECL intensity enhancement was found with no deviation in the Ru(bpy)_3_^2+^ spectrum. A theoretical prediction showed that ECL could be further enhanced when Ru(bpy)_3_^2+^ was closer to the gold array.

Under the condition of ECL measurements, the surface oxide of gold nanomaterials can be formed at a high anodic potential. This surface oxide layer can hinder the electron transfer rate and gradually decrease the ECL intensity, leading to poor stability at repeat measurements. Wilson et al. investigated the ECL scenario generated with single gold nanowire [62]. The as-purchased nanowires did not show any ECL signal due to the block of surface hexadecyltrimethylammonium surfactant. After removing the surfactant and coating the nanowires with a protective polymer blend, gold nanowires showed a better ECL stability because the polymer layer protected the gold surface from electrochemical oxidation and damage. In addition, the polymer thickness significantly influenced the sharpness and reproducibility of ECL images during ECL measurement.

Because the distribution of electrocatalytic activity at single nanomaterials is heterogeneous, it is important to map the ECL signal at the sub-particle level. The uneven electrocatalytic activity on individual 2D gold nanoplates was imaged with submicron resolution [49]. Because of different electrocatalytic rates at different sites, the corners, edges, and flat facet showed a non-uniform ECL distribution. Although much higher ECL intensity was observed at the corners and edges with more defect sites, the flat facet possessed higher ECL stability with time elapsing. To break the optical diffraction limit in ECL microscopy, Chen et al. subsequently develop super-resolution ECL microscopy with a radial fluctuation algorithm (Figure 5c) [48]. The stochastic nature of the ECL emission made the generated photons obey Poisson statistics. The Poisson distribution benefited the super-resolution radial fluctuation algorithm, providing more abundant details on gold nanomaterials with 100 nm spatial resolution. The super-resolution imaging technique provided more detailed electrocatalytic sites on single gold nanospheres, nanorods, and nanoplates. In addition to optical fluctuation imaging methods, single-molecular localization microscopy can also be used to obtain super-resolution image of gold plates. Dong et al. used single-molecule ECL microscopy for mapping chemical activity and reaction kinetics on single gold plates [63]. The high spatial resolution (37 nm) and temporal resolution enabled the trace of dynamic evolution of catalytic sites as a function of time.

## 3. Plasmonic-Based ECL Biosensors

Most plasmonic-coupled ECL mechanisms have been used to fabricate high-performance biosensors due to the enhanced sensitivity and lower limit of detection. Biosensors is a hotspot in the field of ECL, from commercial applications to the frontier of academia. So far, the proposed plasmonic-coupled ECL biosensors can be divided into four categories, although there is a myriad of different types of detection targets. These four categories denote small molecule sensing, genosensing, protein sensing, and cell sensing. In addition, some plasmonic-coupled ECL detection biosensors were performed by ECL microscopy with high spatial and temporal resolution. Therefore, we briefly summarize recent developments concerning these four important groups involving plasmon-coupled ECL biosensors below (Table 1).

### 3.1. Small Molecule Sensing

**Figure 6:**
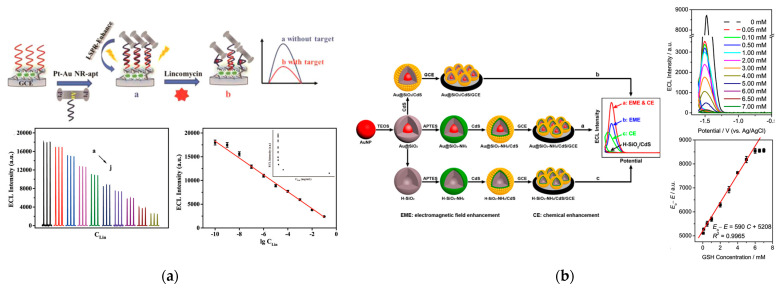
(**a**) Schematic diagram of the SPR-enhanced ECL sensing platform for lincomycin determination. ECL response to different lincomycin concentrations and linear regression curve of the aptasensor for determination of log(lincomycin concentration). Reprinted with permission from *ACS Applied Materials & Interfaces*, Copyright 2022, American Chemical Society [64]; (**b**) Schematic diagram of the dual enhancement ECL system, ECL curves of Au@SiO_2_-NH_2_/CdS/GCE, and a plot of decreased ECL intensity towards GSH of different concentrations. Reprinted with permission from *ACS Applied Materials & Interfaces*, Copyright 2019, American Chemical Society [66].

Lincomycin is a strong bactericide against Gram-positive bacteria; however, it can cause renal dysfunction and increase the drug resistance of Gram-positive bacteria after entering the human body. To detect lincomycin, Li et al. developed a europium metal-organic framework as a scaffold and Au-Pt bimetallic nanorods as a plasma source (Figure 6a) [64]. Because of the plasmon effect of the nanorods, polyaniline-decorated perylene tetracarboxylic dianhydride showed significant enhancement in the ECL signal. Given the superior ECL performance, an ECL aptasensor was designed for the quantitative detection of lincomycin. The detection limit of this aptasensor reached 0.026 ng/mL, which is much lower than the reported analytical methods.

Glutathione (GSH) is an important physiological tripeptide with a thiol moiety. The concentration of GSH in the human body is related to many pathologies such as diabetes, liver disease, and cataracts. To detect GSH with high sensitivity, a new plasma-enhanced ECL sensor was developed. Cao et al. synthesized a nanocomposite using silicon dioxide for controlling the distance between Ru(bpy)_3_^2+^ and AuNPs [65]. Benefiting from a properly controlled distance, ECL had a good interaction with LSPR, leading to the enhanced ECL intensity for the detection of GSH sensitively. The established sensor demonstrated wide response ranges (1.0 fM–1.0 nM and 1.0 nM–1.0 μM) and low detection limit (0.5 fM, S/N = 3) for GSH. Li et al. also discovered a ECL system for sensing GSH by aminated Au@SiO_2_/CdS quantum dots nanocomposites (Figure 6b) [66]. They used two mechanisms for ECL enhancement including LSPR of the Au core triggered by ECL irradiation and electrochemical reactions between modified amino groups and H_2_O_2_. With this dual ECL enhancement strategy, the response mechanism of GSH was also explored.

Due to the severe environmental and human health risks of Microcystins-LR (MC-LR), sensitive MC-LR testing is required. A novel SPR enhanced cathodic ECL biosensor used for MC-LR detection has been designed based on boron and nitrogen co-doped graphene quantum dots (BN-GQDs) [67]. Bismuth nanoparticles (Bi NPs) replace precious metals as the source of SPR, which demonstrated strong mutual enhancement with BN-GQDs leading to the amplification of ECL. In addition to super-sensitive detection for MC-LR, this SPR-ECL sensor can be used in bioanalysis with the advantages of biometrics.

Dopamine (DA) plays a key role in adjusting physiological functions; it is hard to detect in biological samples with electrochemical techniques because of other interfering species. Li et al. reported an enhanced ECL strategy for the analysis of DA under the LSPR-induced enhanced electromagnetic field of Au cores of Au@SiO_2_ NPs [68]. The mechanism of ECL quenched by DA was clearly elaborated and the detection of DA can applied in human serum.

Diclofenac (DCF) is regarded as a new pollutant and has a negative impact on human health. With the use of Au NPs as plasma, Li et al. proposed a transformation mechanism from LSPR to resonance energy transformation (RET) to specifically detect DCF [69]. 3,4,9,10-perylenetetracarboxylic acid–decorated cobalt phosphate (PTCA/CoP) with superior ECL performance benefitted from the special 1D/2D structure was used as the ECL emitter. The transformation of the aptasensor was triggered by the introduction of DCF, which demonstrated specific detection of DCF from 0.1 pM to 10 μM with a low detection limit of 0.072 pM.

### 3.2. Genosensing

**Figure 7:**
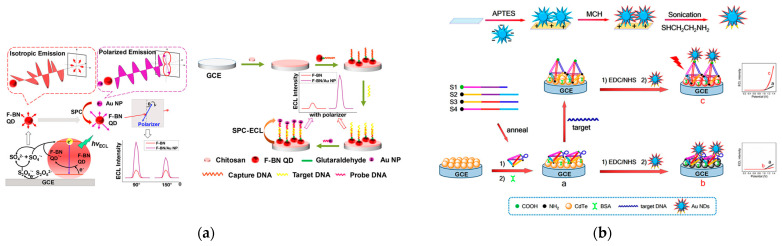

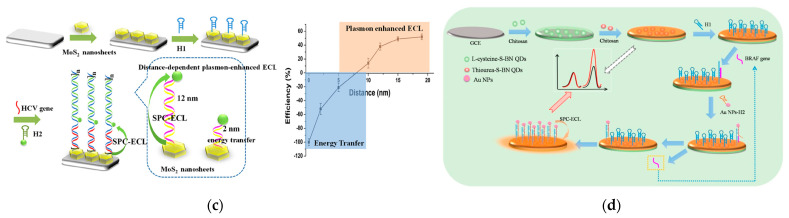
(**a**) Polarized SPC-ECL mechanism of F-BN QDs and schematic illustration of the polarized ECL sensor. Reprinted with permission from *Analytical Chemistry*, Copyright 2020, American Chemical Society [70]; (**b**) Stepwise asymmetric modification for Au NDs and schematic illustration of the LSPR-enhanced ECL sensor based on the DNA tetrahedral nanoswitch. Reprinted with permission from *Analytical Chemistry*, Copyright 2018, American Chemical Society [73]; (**c**) Schematic illustration of the HCR-based sensing process, the relationship between SPC enhanced efficiency, and the distance between MoS_2_ nanosheets and S-BN QDs. Reprinted with permission from *Biosensors and Bioelectronics*, Copyright 2020, Elsevier [74]; (**d**) Schematic illustration of the ECL DNA sensor. Reprinted with permission from *Analytical Chemistry*, Copyright 2019, American Chemical Society [78].

The most widely used plasmonic-based ECL biosensors are genosensors because the hybridization or conformation change of nucleic acids can regulate the distance between ECL emitters and plasmonic structures. Liang et al. first reported the polarized-ECL biosensor for the determination of the K-RAS gene (Figure 7a) [70]. Usually, the ECL emission from luminophores is isotropic so most ECL methods do far have relied on the variation of ECL intensity or wavelength. However, the coupling of ECL emission and surface plasmon can not only result in luminescence signal amplification, but also in a change in the polarized angle of ECL emission. Through the sandwich structure of DNA hybridization, the distance between gold nanoparticles and fluorine-doped BN quantum dots decreased, which caused the SPR coupling effect to ECL polarization. With the assistance of a polarizer between the electrode and detector, polarized ECL was observed and improved the detection sensitivity at a specific polarization angle compared with isotropic ECL signal.

Wang et al. used gold nanobipyramids as a plasmon unit whose horizontal and vertical patterned structures offer different plasmonic properties reflecting on resonance peak positions and polarized absorbance [71]. Accordingly, the ECL polarization of SnS_2_ quantum dots was observed at the gold nanobipyramids patterned structures. Because the ECL polarization was regulated by the orientation, the polarization-resolved ECL was used to analyze miRNA-21 and miRNA-205 expression levels in tumor tissues.

Subsequently, Wang et al. further synthesized gold nanotriangles to enhance the ECL signal by the tip amplification effect [50]. Because of the local surface plasmon resonance and polarization modulation ability of three shape tips, a high-polarization-resolved ECL sensor was fabricated to detect miRNA-221. The SnS_2_ quantum dots were chosen as the ECL emitter whose ECL emission is isotropic. However, in the presence of gold nanotriangles, the ECL generated from SnS_2_ quantum dots showed the characteristics of polarization at the directional angle due to the strong and uniformly distributed hot spot regions. Through T7 exonuclease cutting of the double-stranded hybrid part, the limit of detection of miRNA-221was calculated at 0.71 fM. In addition to using SnS_2_ as a luminophore, Li et al. used SnS_2_ as a plasmonic source for sensing miRNA-21 [72]. Because of the enhancement ECL induced from the SPR effect and the specific recognition of streptavidin (SA), the stable and ultrasensitive quantification of miRNA-21 was achieved in real samples.

To further increase the ECL signal during the recognition process, Li et al. designed gold nanodendrites as a plasmon enhancer due to the extremely strong electromagnetic field located around multiple tip branches (Figure 7b) [73]. Meanwhile, a DNA tetrahedron embedded with a stem-loop hairpin structure acted as a switch to regulate the distance between ECL emitter CdTe quantum dots and ECL enhancer gold nanodendrites. In the absence of targeted DNA, the DNA tetrahedron showed a relaxed state due to the closed hairpin structure. At this time, the ECL signal of CdTe quantum dots was quenched by proximal gold nanodendrites and were in a turned off mode due to the FRET effect. In the presence of targeted complementary DNA, however, the relaxed DNA structure was changed into a rod-like configuration. As a result, the increased distance between CdTe quantum dots and gold nanodendrites caused a significant ECL amplification induced by the LSPR effect. Finally, the concentration of targeted DNA was determined through the configuration changes of DNA structures based on the distance-mediated LSPR-ECL system.

In addition to a noble metal as the plasmonic source, Liu et al. developed a plasmon-enhanced ECL sensor using semiconductor MoS_2_ nanosheets and sulfur doped boron nitrogen quantum dots (Figure 7c) [74]. Because of the distance-dependent plasmon effect, variable lengths of DNA strands were employed to control the distance between the two. With the gradual increase in the distance, the energy transfer efficiency was weakened but the surface plasma coupling effect was strengthened. After controlling the subtle distance, a hybridization chain reaction amplification was employed to determine the hepatitis C virus gene.

Lu et al. used hollow gold nanocages as SPR nanostructures for the detection of DNA [75]. The ECL biosensor included Ru(bpy)_3_^2+^ doped silica nanoparticles (RuSi NPs) as the ECL donor and tetrahedron DNA as the scaffold to regulate the distance between the ECL donor and emitter pairs. Given that the plasmon absorption spectrum of hollow gold nanocages overlapped the ECL emission spectrum of RuSi NPs, the ECL energy transfer between the two can occur to a great extent. In addition, a cyclic DNA amplification strategy was employed to increase the binding amount of gold nanocages as well as the detection sensitivity. As a result, after the miRNA-141 was introduced into the solution, the binding of miRNA-141 in the ECL biosensor made the gold nanocages close to RuSi NPs. The decreasing signal values revealed a good linear relationship with the concentration of miRNA-141.

Kitte et al. proposed a surface-plasmon enhanced ECL strategy with Au NPs as the plasma source [76]. To realize the superior ECL signals, nitrogen vacancy-modified g-CN (NVCN) was used as the ECL emitter, which showed better ECL performance than g-CN. The problem of electrode passivation was avoided because of trapping the excess electrons in the NV sites. Based on the improvement of the ECL emitter and the SPR action from Au NPs, the established surface-plasmon enhanced aptasensor demonstrated superior responses to miRNA-133a with a limit of detection (LOD) of 0.87 aM.

Liu et al. designed a visualized ECL sensor by the switch between resonance energy transfer quenching and surface plasmon resonance enhancing for the detection of the Shiga toxin-producing Escherichia coli gene [77]. The DNA sensor employed a hairpin structure, which connected a BN quantum dot and a gold nanoparticle at the ends. In this case, the ECL intensity of the BN quantum dot was quenched by the adjacent gold nanoparticle due to the energy transfer effect. When the target DNA was identified by the hairpin DNA, the hairpin structure transformed to a rigid linear structure, and the distance between the two entities increased. Thus, the SPR effect became dominant and the ECL intensity of the BN quantum dots significantly improved. This detection mode possessed high selectivity, stability, and sensitivity to quantify the Escherichia coli gene. Because of the satisfactory ECL intensity of the BN quantum dots, a household smartphone was able to record visual ECL signals from the DNA sensor.

Liu et al. synthesized various boron nitride quantum dots by precise control and regulation with sulfur precursors such as thiourea and L-cysteine (Figure 7d) [78]. Because the two kinds of S-regulated boron nitride quantum dots possessed different ECL wavelengths, a ratiometric and enzyme-free ECL sensor was designed with the assistance of the amplified surface plasmon-coupled ECL strategy. The ECL sensor was ultimately used to detect the BRAF gene by the so-called target-catalyzed hairpin assembly (CHA) amplification strategy. The dual-wavelength ECL sensor used quantum dots with a 535 nm ECL wavelength as a reference and quantum dots with a 620 nm ECL wavelength as an analytical tag. When gold nanoparticles approached one of the quantum dots (620 nm emission) through the DNA hairpin, the ECL emission near the gold nanoparticles underwent resonant interactions and signal amplification. The ratiometric signal can quantify the BRAF gene from 1 pM to 1.5 nM.

Liang et al. proposed that surface plasmon-coupled ECL can improve ECL performance with polarization characteristics [79]. Based on this discovery, a novel polarization-resolved sensor was used as a sensitive multiple measurement method for breast cancer (BRCA) susceptibility genes. The emission wavelength of the luminophore coincided with the plasmon resonance wavelength of Au NPs and gold-coated silver nanoparticles, which realized the ECL enhancement with effective coupling between the surface plasmons of the nanometal and the emission of the luminophore. In addition, the polarization characteristics converted the isotropic ECL signals into polarization, realizing the ultrasensitive detection for BRCA1 and BRCA2 simultaneously.

Lu et al. developed a gold inverse opal plasmonic array, which produced a high electromagnetic field at the inner surface [80]. The high electromagnetic field enhanced the ECL emission of Ru(bpy)_3_^2+^-doped silica nanoparticles. After comparing the ECL signal from different gold electrode surfaces, it was found that gold inverse opal significantly improved the ECL intensity of Ru(bpy)_3_^2+^-doped silica nanoparticles through the strong electromagnetic field. The outperforming electrode substrate showed a good sensitivity and selectivity for the determination of miRNA-21.

Cumba et al. used nanocavity arrays tuned by varying the cavity size through nanosphere lithography [81]. Using the nanosphere template and electrodeposition, a 3D gold array with a broad cavity plasmon was designed to enhance the overall ECL intensity. The 3D electrode arrays, with a pore diameter ranging from 240 nm to 2 µm, offered significant merits in both conventional and bipolar ECL for the detection of target DNA sequences associated with methicillin-resistant Staphylococcus aureus. The analytical sensitivity on the basis of the gold nanocavity electrode was improved approximately 7-fold than that observed at a planar electrode.

Chen et al. used the SPR enhanced ECL of CuZnLnS quantum dots as the foundation and thus designed an ultrasensitive sensor for the epidermal growth factor receptor (EGFR) gene via DNA hybridization reactions [82]. Three kinds of CuZnLnS quantum dots with different stabilizers (mercaptopropionic acid, L-glutathione, cysteamine) were compared in terms of the ECL efficiency. It was found that mercaptopropionic acid capped CuZnLnS quantum dots showed the strongest ECL intensity. In the presence of gold nanoparticles, the detection limit of this sensor reached 0.0043 nM and linear relationship ranged from 0.05 nM to 1 nM.

To detect the Kirsten rat sarcoma (K-RAS) gene, a mouse sarcoma virus oncogene, Zhang et al. developed a sandwich-typed sensor by combining magnetic-plasmonic Fe_3_O_4_ yolk/Au shell and g-C_3_N_4_ quantum dots [83]. Because the middle cavity in the yolk-shell structure eliminated the electron transfer between Fe_3_O_4_ nanoparticles and the Au shell, the yolk-shell structure showed a strong surface plasmon coupling effect to enhance the ECL intensity of g-C_3_N_4_ quantum dots. As a result, the sandwich sensor exhibited excellent analytical performance for DNA determination. The surface plasmon enhanced ECL effect is restricted by the spectral overlap between the ECL emission and the absorption band of Au NPs. To provide a further discussion of wavelength-dependent plasmon enhanced ECL, Zhang et al. synthesized sulfur-doped graphite phase carbon nitride QDs to increase ECL efficiency [84]. This wavelength-dependent biosensor provided proper selectivity for K-RAS detection.

It is essential to control the distance between the ECL emitter and plasmonic enhancer such as Au NPs in the surface plasmon-enhanced ECL system. Feng et al. proposed a method to detect miRNA-21 using DNA templated silver nanoclusters as the ECL emitter and AuNPs as the source of LSPR [85]. By regulating proper distances between the luminophore and Au NPs, and the appropriate size of Au NPs controlled by the electrodeposition time, the optical LSPR enhanced ECL strategy for the detection of miRNA was developed with a high sensitivity of 0.96 aM. Yang et al. developed a direct and rapid method for ctDNA detection based on the ECL-RET strategy [86]. The introduction of hairpin DNA probes modified with Au NPs on the surface of the electrode results in a decrease in the ECL signal because of ECL-RET. After target DNA molecules anneal to Au NPs probes, the increased distance between Au NPs and QDs contributes to the increased ECL. Compared with conventional biosensors for ctDNA, the proposed ECL-RET-based biosensor had more potential in practical biosensors.

### 3.3. Protein Sensing

Prostate-specific antigen (PSA) has clinical significance as a biomarker of prostate cancer. However, the existing problems for detecting PSA include complex pretreatments, poor sensitivity, and low selectivity, thus limiting the development of PSA biosensors. Li et al. fabricated insulating gold nanoparticles with silica shells to control the plasmonic coupling effect [87]. By adjusting the silica-shell thickness and gold nanoparticle size, the plasmonic enhancement effect was maximized. As a result, a 2D ordered nanomembrane on an ITO surface was designed and found to be able to improve the ECL signal over 1000-fold compared with the classical Ru(bpy)_3_^2+^-tripropylamine ECL system. The fabricated ECL probe was used as an immunosensor, showing a very low detection limit for PSA with a sandwich-type design.

**Figure 8:**
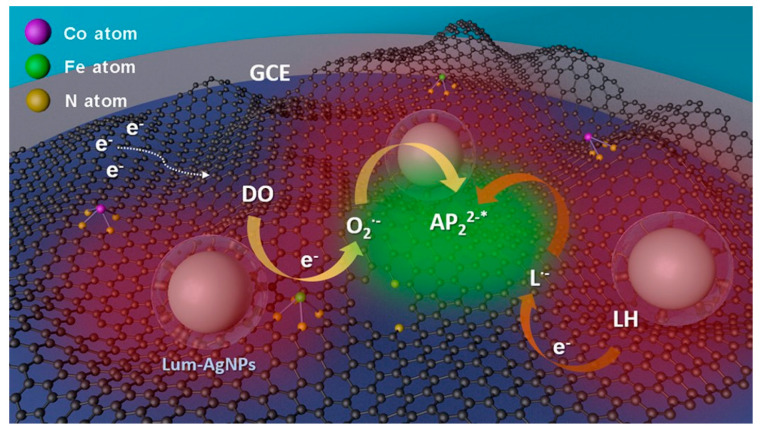
Proposed signal enhancement mechanism of the Lum-AgNPs@Fe,Co D-SAC-based luminol-DO ECL system. Reprinted with permission from *Analytical Chemistry*, Copyright 2022, American Chemical Society [88].

Bushira et al. developed a plasmon-boosted ECL system with Ag nanoparticles (Figure 8). The ultrasensitive detection of PSA was achieved through the use of dissolved O_2_ as the co-reactant and dual-active metal sites as catalysts [88]. This PSA immunosensor demonstrated a wide detection range of 1 fg/mL to 1 μg/mL and an ultrasensitive detection ability of 0.98 fg/mL. Liu et al. used the ECL-RET strategy to develop a biosensor for PSA [89]. To establish the ECL-RET platform, Ag NPs were used as the acceptor for ECL-RET and two-dimensional planar semiconductor nanomaterials of graphitic carbon nitride quantum dots were selected for use as the donor. Because of the great overlap between the emission spectrum of materials and the absorption band of Ag NPs, the proposed sensor had a good performance for RET. In addition to the RET effect, the introduction of two co-reactants also contributed to amplification of the ECL. This strategy indicated a novel method for PSA quantization. Wang et al. fabricated a biosensor with an antibody–antigen sandwich structure for specific determination of PSA [90]. To generate and amplify the ECL signal, they synthesized a composite nanomaterial contained Au-SiO_2_ doping with Ru(bpy)_3_^2+^. This LSPR enhanced ECL sensor was applied in a sample with a low concentration of PSA.

To compare three different detection modes (quenching method, amplification method, ratiometric method) to determine thrombin, Isildak et al. used CdS nanocrystals as the luminophore and gold nanoparticles as the acceptor through aptamer–thrombin interaction [91]. The first comparison between the amplification method and quenching method showed a more sensitive signal from the “turn-on” recognition mode. This is because the plasmonics on gold nanoparticles enhance the ECL emission of CdS quantum dots by regulating a suitable distance between the two. However, the ratiometric method showed the greatest sensitivity when the ECL intensity of CdS declined; however, that of luminol increased in the presence of thrombin. The unique point of this work is to compare three different ECL methods for the determination of thrombin.

### 3.4. Cells Sensing and Microscopy

**Figure 9:**
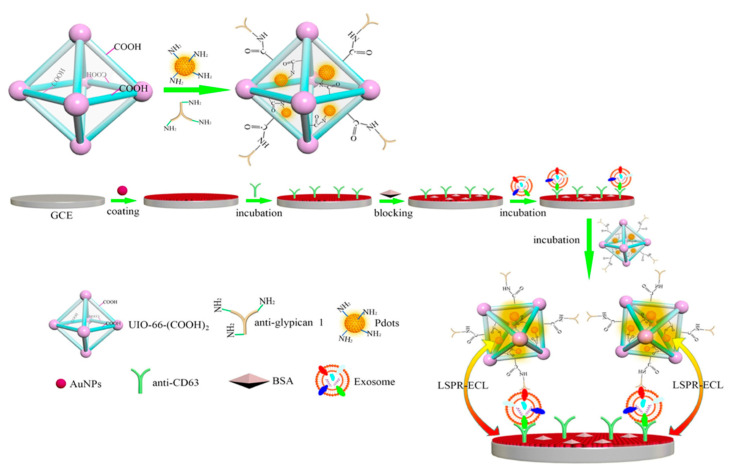
The schematic diagram of ECL sensing for pancreatic exosome detection. Reprinted with permission from *Analytical Chemistry*, Copyright 2022, American Chemical Society [92].

Exosomes are important biomarkers in many biological processes because they contain considerable molecular information, including nucleic acids and proteins. After being released from cells, exosomal proteins become a novel class of diagnostic biomarkers for cancer diagnosis in the clinic. Xiong et al. fabricated a surface plasmon-coupling ECL immunosensorusing polymer dots (Pdots) as emitters and gold nanoparticles as enhancers (Figure 9) [92]. The hot electrons of the gold nanoparticles were excited by the ECL light of Pdots, and thus reached the surface plasmon states, which, in reverse, enhanced the ECL intensity of Pdots. The advanced immune-biosensor showed high selectivity for the exosomes from different cell lines such as PANC-01, HeLa, MCF-7, and HPDE6-C7.

Han et al. developed a localized surface plasmon-enhanced ECL biosensor using urchin-like gold and silver nanoparticles for bacteria detection [93]. Because the ECL emission from oxygen (478 nm) overlapped with the surface plasmon band of urchin-like Au and Ag nanoparticles, the ECL signal was enhanced significantly. After optimization of the reaction conditions of the biosensor, a wider linear range and lower detection limit were obtained. In addition, the biosensor showed good versatility to detect three species of bacteria and biological macromolecules.

Wang et al. discovered that antibody molecules and cells can hinder the ECL quenching effect of gold nanoparticles, whose hot electrons hampered co-reactant 2-(dibutylamino)-ethanol (DBAE) to give off electrons [94]. First, nitrogen doped molybdenum oxynitride nanotube arrays acted as ECL emitters. After modification of the gold nanoparticles on this nanotube array, the SPR effect of the gold nanoparticles quenched the ECL intensity. However, the immobilization of gold nanoparticles acted as the linker via the affinity effect between gold and nitrogen moieties on antibody molecules, which also led to a slight increase in the ECL signal due to the barrier effect. Because the antibody molecules specifically recognized HepG2 cells, the ECL recovery degree revealed the concentration of cells.

Zhou et al. developed a dual-potential ratiometric ECL biosensor for cancer cells, such as Ramos cells, which used two types of nanomaterials including Au@luminol nanocomposite and CdS nanocrystal as ECL emitters independently [95]. First, Au nanosucculent films electrodeposited on the working electrode increased the effective area of the electrode to assemble DNA and improved the conductivity of the sensing interfaces. In addition to the improvement of electrochemical properties, SPR originating from Au NPs also promoted the ECL intensity after the formation of the reticulate structure by alternate hybridization with DNA-modified CdS nanocrystals. This developed dual-potential ECL sensor had the ability to identify Ramos cells with a LOD of 20 cells/mL and had potential for the detection of other types of cancer cells.

Because gold nanoparticles possess good conductivity, cell biocompatibility, and easy modification, they have been widely used as plasmonic enhancers to construct visualized biosensors. Cao et al. synthesized heterogeneous Ru(bpy)_3_^2+^@SiO_2_/Au as functional nanoprobes [96]. A closed bipolar electrode configuration was fabricated to avoid the chemical interference between sensing and reporting reactions. Thanks to gold nanoparticles with high conductivity and an active surface area, the ECL intensity was significantly enhanced. The nanoprobe specifically targeted PSA on HeLa cell membranes, leading to visualized detection of tumor markers on single cells. In addition, the Ru(bpy)_3_^2+^@SiO_2_/Au nanoprobe was also used to realize single-biomolecule imaging for the first time [97]. Because of the high ECL signal of the Ru(bpy)_3_^2+^@SiO_2_/Au nanoprobe, a single cytokeratin 19 biomarker was recognized by the monoclonal antibody against the cytokeratin 19 modified Ru(bpy)_3_^2+^@SiO_2_/Au nanoprobe. As a result, the direct observation of single biomolecules at the electrode surface as well as the cellular membrane demonstrated that the local confinement enhancement effect enabled spatially resolved imaging of single proteins.

To boost the ECL signal for improved imaging sensitivity, Chen et al. developed a synergistic co-reactant that contained guanine-rich ssDNA loaded high-index faceted gold nanoflowers [98]. The gold nanoflowers acted as not only a carrier for guanine-rich ssDNA, but also an ECL enhancer. The synergistic co-reactant reached the highest ECL enhancement factor of up to 234 times, outperforming other nano-co-reactants with single components. Through the catalytic route ECL reactions, the gold nanoflower/G-ssDNA hybrid with the carcinoembryonic antigen (CEA) aptamer enabled selective recognition of CEA on the membrane of human breast adenocarcinoma (MCF-7) cells.

## 4. Conclusions and Future Perspectives

In this review, we first introduced the concept related to the construction of the plasmon-coupled ECL phenomenon and the mechanisms of plasmonic ECL. Under the influence of plasmonic effects, the plasmon-coupled ECL responded in two aspects, including the absolute intensity and polarization angle of ECL. The quenched ECL signal is based on the plasmon effect of resonant energy transfer and the ECL enhancement, because of surface plasmon resonance enhancement, relies on the adjustment of the distance between ECL emitters and plasmonic materials. Thanks to the effect of plasmon, the plasmon-coupled ECL had performed well with a high sensitivity and the low detection limit, which can have good applications in the establishment of biosensors. Accordingly, we also summarized the plasmon-coupled ECL biosensors for the detection of small molecules, genes, proteins, and cells. The key elements in the plasmon-coupled ECL biosensor are the construction of plasmonic materials and regulated scaffolds for highly sensitive and selective analysis. Because of the enhancement of plasmonic materials, single particle and cell imaging is accomplished by ECL microscopy, which in turn demonstrated the heterogeneous plasmonic effect in single particles.

In terms of future perspectives, although the plasmon-coupled ECL has been well applied in sensing, the mechanisms of coupling ECL and the plasmon need to be understood further. The current plasmon-coupled ECL also has challenges in commercial applications because of complicated surface functionalization process, the biological toxicity of the ECL emitter, and so on. Nowadays, the fabrication of plasmonic materials can be easily achieved in the laboratory. However, the repeatability of synthesizing even plasmonic materials at an industrial scale is still a big challenge. In addition to controlling the distance between luminophores and plasmonic materials, properties of the materials, such as morphology, also play important roles in plasmonic properties (optical, electronic, and magnetic properties). Developing a plasmon-coupled ECL strategy with non-precious metals as plasmonic producers and bio-friendly ECL emitters requires more attention in future developments. In addition, recent advanced ECL microscopy with good spatial and temporal resolution can be used as a powerful tool to provide fruitful insights into the fundamental mechanisms in plasmon-coupled ECL that promotes single-molecule imaging techniques. This combination between a microscopy strategy and plasmon-coupled ECL also can provide more information to screen for better plasmonic materials for ultrasensitive ECL biosensors. Because ECL is a light source-free analytical technique, the mechanisms used in plasmon-coupled fluorescence or Raman scattering cannot be directly used in plasmon-coupled ECL biosensors. Therefore, ECL microscopy provides a visual tool to investigate the effect of plasmon-coupled ECL, thereby offering a direction for optimization of the synthesis route of plasmonic materials. We believe that more plasmonic-based ECL biosensors can be constructed with the development of plasmonic materials. Learning from other realms of plasmonic optics, plasmonic based ECL biosensors could make a big step towards commercial applications.

## Figures and Tables

**Table 1:** Summary of recently proposed plasmonic-based ECL biosensors according to different types of analysts.

	Targets	Plasmonic Materials	LOD	Linear Range	Ref
Small molecule	Lincomycin	Au-Pt bimetallic nanorods	0.026 ng/mL	0.1 mg/mL–0.1 ng/mL	[64]
	Glutathione	Au nanoparticles	0.5 fM	1.0 fM–1.0 nM	[65]
				1.0 nM–1.0 μM	
		Au cores (ca. 55 nm)	0.065 mM	0.10–6.00 mM	[66]
	Microcystins-LR	Bismuth nanoparticles	0.003 pM	0.01–5000 pM	[67]
	Dopamine	Au core (ca. 68 nm)	0.004 μM	0.01–600 μM	[68]
	Diclofenac	Au nanoparticles	0.072 pM	0.1 pM-10 μM	[69]
Gene	K-ras gene	Au nanoparticles	0.03 fM	0.1 fM–10 nM	[70]
	miRNA-21	Au nanobipyramids	3.3 fM	0.01 pM–10 nM	[71]
	miRNA-205		1.7 fM	5 fM–1 nM	
	miRNA-221	Au nanotriangle	0.71 fM	1 fM–1 nM	[50]
	miRNA-21	SnS_2_ nanoplates	0.6 aM	n.r.^a^	[72]
	nucleic acid	Au nanodendrites	30 aM	1.0–500 fM	[73]
	Hepatitis C virus gene	MoS_2_ nanosheets	0.17 pM	0.5 pM–1 nM	[74]
	miRNA-141	Au nanocages	0.4 fM	1.0 fM–10 pM	[75]
	miRNA-133a	Au nanoparticles	0.87 aM	1 aM–100 pM	[76]
	Shiga toxin-producing Escherichia coli gene	Au nanoparticles	0.3 pM	1 pM–5 nM	[77]
	BRAF gene	Au nanoparticles	0.3 pM	1 pM–1.5 nM	[78]
	Breast cancer-related genes (BRCA1, BRCA2)	Au nanoparticles, gold-coatedsilver nanoparticles	n.r.^a^	100 aM–1 nM	[79]
	miRNA-21	Gold inverse opals	3.3 fM	5.0 fM–5.0 pM	[80]
	MRSA DNA	3D Au array	1 μM	10 nM–30 μM	[81]
	Epidermal growth factor receptor gene	Au nanoparticles	0.0043 nM	0.05 nM–1 nM	[82]
	K-RAS gene	gold shell	0.3 fM	1 fM–1 nM	[83]
		Au nanoparticles	16 fM	50 fM–1 nM	[84]
	miRNA 21	Au nanoparticles	0.96 aM	1 aM–10^4^ fM	[85]
	Circulating tumor DNA	Au nanoparticles	0.0023 fM	0.01 fM–1 pM	[86]
Protein	Prostate-specific antigen	Au nanoparticles	3 fg/mL	10 fg/mL–1 μg/mL	[87]
		Ag nanoparticles	0.98 fg/mL	1 fg/mL–1 μg/mL	[88]
			0.0046 pg/mL	1 × 10^−5^–500 ng/mL	[89]
		Au nucleus	7 × 10^−7^ ng/mL	n.r.^a^	[90]
	Thrombin	Au nanoparticles	92 pg/mL	500–5000 pg/mL	[91]
			6.5 pg/mL	50–1000 pg/mL	
			500 fg/mL	5–500 pg/mL	
Cell	Pancreatic cancer exosomes	Au nanoparticles	400 particles/mL	1.0 × 10^3^–1.0 × 10^6^ particles/mL	[92]
	S. aurenus	Urchin-like Au and Ag nanoparticles	52 CFU/mL	2 × 10^2^–2 × 10^8^ CFU/mL	[93]
	HepG2	Au nanoparticles	47 cells/mL	50–13800 cells/mL	[94]
	Ramos	Au nanoparticles	20 cells/mL	80–8 × 10^5^ cells/mL	[95]
	PSA on LNCaP	Au nanoparticles	3.0 pg/mL	10 pg/mL–50 ng/mL	[96]
			31 pg/mL	0.05–50 ng/mL	
	Cytokeratin 19 on MCF-7	Au nanoparticles	0.12 pg/mL	0.01–10 ng/mL	[97]
	CEA on MCF-7	High-index faceted gold nanoflower	n.r.^a^	n.r.^a^	[98]

^a^ not reported.

## Data Availability

Not applicable.
